# Medical AI in the Americas: policy, validation, and representativeness

**DOI:** 10.1016/j.lana.2026.101579

**Published:** 2026-07-15

**Authors:** The Lancet Regional Health – Americas

A medical artificial intelligence (AI) model can perform well in a development study and still fail patients. Tools built to support diagnosis, triage, risk prediction, or treatment decisions can become inaccurate, or unsafe, when deployed in populations and health systems different to those in which they were trained and tested. As Cruz-Suarez and colleagues argue, AI will extend evidence-based medicine only if it is governed by transparent validation, post-deployment monitoring, and data that represent the patients it is meant to serve. Across the Americas, transparent validation and post-deployment monitoring remain inconsistent, data are uneven, and the populations most likely to benefit from safer, more affordable innovation are often the least visible in the evidence base.

Evidence on medical AI in the Americas is constrained by the fact that many datasets do not describe or include the populations in which these systems might ultimately be used. Of 181 datasets in the widely used PhysioNet repository, less than 7% reported race, ethnicity, sex or gender, and age together; most participants were male, and most studies were done in North America. Furthermore, across low-income and middle-income countries, limited infrastructure compounds scarce: one review found that only 7.4% of countries had a national AI strategy, more than 60% of models relied on non-representative datasets, and less than 10% of institutions offered structured AI training.

Performance metrics from development or regulatory submissions can hide clinically important differences in safety and validity across populations. A model's apparent confidence can conceal bias, and systems trained largely in high-income, data-rich settings can fail under-represented populations in Latin America and the Caribbean. A review of 692 machine-learning-enabled or AI-enabled medical devices cleared by the US Food and Drug Administration between 1995 and 2023 found that only 3.6% reported race or ethnicity and 99.1% provided no socioeconomic data, 1.9% were linked to a publication reporting safety and efficacy, and 9.0% included prospective post-market surveillance.

Policy responses are beginning to emerge, although with different levels of specificity, enforceability, and relevance to health. The EU's AI Act classifies many medical AI applications as high risk and links them to conformity and monitoring requirements. In the USA, federal policy has shifted towards a uniform, minimally burdensome national framework, including efforts to prevent a fragmented state-by-state approach. In Latin America, Peru became one of the first countries in the region to adopt a general AI framework, with implementing regulation approved in 2025. Brazil's Bill 2338/2023 has cleared the Senate and remains before the Chamber of Deputies. Several countries still have no national AI plan. Together, these developments show regulatory activity, but no coherent health-specific approach to medical AI.

Legislation will matter only if it changes the evidentiary standard for model development and use. Regulation should be paired with national strategies, representative external validation, open data, and open science. A review of health-related open datasets in Latin America identified only 61 open datasets across 23 countries, most drawn from Brazil's DATASUS health information database. This is a narrow foundation for a region of profound demographic, epidemiological, and health-system diversity. Stronger regional data-sharing frameworks, common formats, privacy-preserving infrastructure, and shared validation standards would embed representativeness in dataset construction, model development, and evaluation.

As countries in the Americas define their approaches to AI governance, the opportunity is to make clinical validity, local performance, and post-market accountability conditions of use rather than later additions. The region now needs rules that require evidence of these conditions before systems are procured, deployed, or scaled. Implementation is a challenging aspect as many regulators in the Americas lack the capacity, data access, and infrastructure to audit AI after deployment. Whether emerging frameworks and policies converge or fragment will determine whether medical AI improves diagnosis, risk prediction, triage, and access to decision support across the Americas, or deepens existing inequities in who is represented, tested, and safely served. As this work unfolds, *The Lancet Regional Health – Americas* will continue to support rigorous regional research and debate on AI and digital health. Medical AI must be built for all people in the Americas, not only for those already counted in the data.
